# Comparison of *Pneumocystis* nucleic acid and antibody profiles and their associations with other respiratory pathogens in two Austrian pig herds

**DOI:** 10.1371/journal.pone.0185387

**Published:** 2017-09-25

**Authors:** Christiane Weissenbacher-Lang, Nora Nedorost, Christian Knecht, Isabel Hennig-Pauka, Mathias Huber, Thomas Voglmayr, Herbert Weissenböck

**Affiliations:** 1 Institute of Pathology and Forensic Veterinary Medicine, Department of Pathobiology, University of Veterinary Medicine Vienna, Vienna, Austria; 2 University Clinic for Swine, Department for Farm Animals and Veterinary Public Health, University of Veterinary Medicine Vienna, Vienna, Austria; 3 Traunkreis Vet Clinic, Ried im Traunkreis, Austria; The University of Melbourne, AUSTRALIA

## Abstract

*Pneumocystis carinii* f. sp. *suis* (PCS) nucleic acid and antibody profiles on two Austrian-farrow-to-finish farms were investigated. Furthermore, associations with other respiratory pathogens were evaluated. Respiratory specimen and sera from pigs of five age classes between the 1^st^ week and the 3^rd^ month of life as well as samples from sows were analyzed. On Farm A, PCS infection occurred early in life. The suckling piglets were already infected in the 1^st^ week of life and the pigs remained positive until the 3^rd^ month of life. On Farm B, pigs were infected later, between 3 and 4 months of age. The maximum PCS nucleic acid load on Farm A was 8.3 log10 genome copies/mL BALF, whereas on Farm B the PCS burden was significantly lower, with 4.0 log10 genome copies/mL BALF. Anti-PCS antibodies were detected in sows, as maternal antibodies in suckling piglets and as an immunological reaction to infection. On both farms, PCS infection was accompanied by several co-infections. On Farm A, there were concurrent infections with PRRSV, a virulent strain of *Haemophilus parasuis*, and *Mycoplasma hyopneumoniae*. On Farm B, PCS was accompanied by infections with swine influenza virus, *Mycoplasma hyopneumoniae*, and a non-virulent strain of *Haemophilus parasuis*. The results clearly show that the PCS profiles can vary between farms. Younger pigs may be more susceptible as they had higher PCS burdens. It is possible that PCS may contribute to a respiratory disease in pigs and further investigation of its potential role is warranted.

## Introduction

*Pneumocystis* spp. are a group of highly diversified opportunistic fungi which are adapted to the lungs of a large variety of mammals [1]. The number of *Pneumocystis* carriers within a population increases under immunosuppressive circumstances and the affected patients develop severe interstitial pneumonia. Furthermore, infection with *Pneumocystis* is suggested to be an important co-factor in the progression of some pulmonary diseases [2].

In pigs with pneumonia, *Pneumocystis carinii* f. sp. *suis* (PCS) occurs with a relatively high frequency of 51% [3]. As in some pig production units different age classes may be kept together in one facility and thus share the same microbiological environment, these animals are predisposed to polymicrobial caused diseases. With continuous flow of the pigs through the system a steady transmission of respiratory pathogens occurs from sows to piglets and from older to younger pigs. Additionally, the risk of a herd contracting respiratory problems increases with the number of animal groups introduced and the number of different sources of the animals [4]. Respiratory diseases in pigs are common in modern pig production worldwide and are one of the main reasons for economic losses. Porcine respiratory diseases are polymicrobial and multifactorial in nature and result from infection with various combinations of primary and secondary respiratory pathogens, as well as from non-infectious factors [5]. Generally, one pathogen acts as key agent for secondary invaders by lowering the local and sometimes also the systemic defence mechanisms of the host. The pathogens involved can vary considerably between different production sites. PCS can frequently be detected in pigs co-infected with other respiratory pathogens [3,6]. In these previous studies, only single lung tissue samples were investigated, and the results did not allow any conclusions to be drawn about the epidemiology of the various pathogens within a production site. The presence of PCS in pigs of different age classes on farm level, as well as possible interactions with concurrent co-infections have not yet been illuminated.

The aim of the present cross-sectional study was to evaluate the levels of PCS nucleic acid and antibody in two Austrian farrow-to-finish farms with onset of respiratory symptoms in different age classes. In addition, their associations with porcine circovirus type 2 (PCV2), porcine reproductive and respiratory syndrome virus (PRRSV), swine influenza virus (SIV), *Haemophilus parasuis* (HPS), and *Mycoplasma hyopneumoniae* (MH) were investigated.

## Materials and methods

Bronchoalveolar lavage fluid (BALF), oral fluid and serum samples of pigs from different age classes (suckling piglets in the 1^st^ week of life (S1), suckling piglets in the 3^rd^ week (S2), weaned piglets in the 2^nd^ month (W1), weaned piglets in the 3^rd^ month (W2), fattening pigs in the 4^th^ month (F), and sows (SOW)) on two Austrian farrow-to-finish farms (size of Farm A: 45 sows/250 fattening pigs, size of Farm B: 160 sows/800 fattening pigs) with chronic respiratory disorders either in suckling piglets (Farm A) or at 2 months of age (Farm B) were collected for routine testing. Sows on both farms were routinely vaccinated against porcine parvovirus and erysipelas. Piglets on Farm A and B were vaccinated twice against MH in the 1^st^ and 4^th^ weeks of life and vaccinated once against PCV2 in the 4^th^ week of life. On Farm A, 45 BALF (S1: 8, S2: 7, W1: 8, W2: 8, F: 14) and 42 oral fluid samples (S1: 4, S2: 7, W1: 8, W2: 8, F: 15) were collected. On Farm B, 47 BALF (S1: 3, S2: 13, W1: 8, W2: 8, F: 15) and 35 oral fluid samples (S1: 1, S2: 6, W1: 8, W2: 7, F: 13) were collected. On each farm, 63 serum samples were collected (S1: 8, S2: 8, W1: 8, W2: 8, F: 15, SOW: 16). From sows only serum samples were collected. For sample collection, all pigs except the sows were anesthetized with a combination of 2 mg of azaperone per kg body weight and 10 mg of ketamine per kg body weight, both administered intramuscularly. For every pig, sterile collection equipment was used, and the samples were handled carefully, and processed under sterile conditions preventing sample-to-sample cross-contamination. For the collection of the BALF samples the pigs were placed in a ventral recumbency, with the sternum on the midline. The forelimbs were stretched back beside the thoracic wall against the abdomen, the hindlimbs were placed in a neutral position to ensure a straight and stable orientation of the spinal column. The head was elevated until the mouth was positioned along the dorsal line. A flexible tube was inserted into the trachea using a laryngoscope and pressed against the hard palate. Sodium chloride (approx. 20 mL) was instilled through the flexible tube and collected by aspiration. The aspirated samples were centrifuged at 2,000 × g for 10 min. The supernatant was discarded and the pellet was resuspended in phosphate buffered saline (PBS). Oral fluid samples were collected by centrifugation of oral swabs that had been placed into the mouths of the pigs for several minutes. Blood was collected by venipuncture of the anterior vena cava or jugular vein and centrifuged at 2,000 × g for 10 min. BALF pellets, oral fluid, and serum samples were stored at –20°C until analysis. As the samples were collected for routine diagnostic purposes and not specifically for this study, and as the farm owners’ consent to use the samples and data were obtained in advance, no ethics committee approval was required. The sampling methods were either non-invasive (oral fluid samples) or carried out in a way that ensured the welfare of the pigs (BALF and serum samples). Compliance with the Austrian federal law for animal experiments [7] and good scientific practice [8] was ensured.

PCS was detected by qPCR, PCV2, HPS and MH by conventional PCR, and PRRSV and SIV by conventional reverse transcriptase PCR (RT-PCR). Examination of BALF samples for other respiratory agents, including *Actinobacillus pleuropneumoniae* or *Pasteurella multocida* could not be carried out because of the limited amount of DNA extractable from BALF. Detection of microorganisms was prioritized according to the clinical and serological findings on the farms. Primer and probe sequences and sample analysis matrix are presented in Table 1. As HPS is ubiquitous and serovars differ in their pathogenicity and virulence, 4 samples per farm from different age classes were submitted for Sanger DNA sequencing. Antibody titres against *Pneumocystis* were determined by XpressBio *Pneumocystis carinii* swine ELISA (Express Biotech International, Frederick, MD, USA), which was performed according to the manufacturers’ specifications. Samples with an optical density (OD) of > 0.3 were regarded as positive.

**Table 1:** Primer and probe sequences and the sample analysis matrix.

Pathogen	Sample matrix	Method	Genomic region	Size (bp)	Sequences	Reference
***Pneumocystis carinii* f. sp. *suis***	BALF	qPCR	mtLSU rRNA	315	fw: 5′-TAGCTGGTTTTCTGCGAAAT-3′; rv: 5′-TTCTGGGCTGTTTCCCTTTA-3′; probe: 5′ FAM-ATCTCAGAATGGCTAATAAGTTAGG-3′ TAMRA	[9]
**PCV2**	serum	PCR	capsid protein gene	101	fw: 5′-GGTACTCCTCAACTGCTGTCC-3′; rv: 5′-GGGAAAGGGTGACGAACTGG-3′	[10]
**PRRSV EU+US**	serum	RT-PCR	ORF5	204	fw: 5′-TYCAATCAAGGCGCWGGRAC-3′; rv: 5′-TCGCCCTAATTGAATAGGTG-3′	[6]
**SIV**	oral fluid	RT-PCR	nucleocapsid protein gene	327	fw: 5′-CTGGGCTATAAGAACCAGGA-3′; rv: 5′-TCTGCATTGTCTCCGAAGAA-3′	this study
***Haemophilus parasuis***	BALF	PCR	outer membrane protein gene	165	fw: 5′-TGATGGTCAATTGCGTCT-3′; rv: 5′-CGAGTCTCATAACGACCAAA-3′	[11]
***Mycoplasma hyopneumoniae***	BALF	PCR	16S rRNA	126	fw: 5′-aaagcgtccgtaggtttttt-3′; rv: 5′-TATTCCACCACTCCACTAGG-3′	[3]

bp = PCR product size in base pairs, fw = forward primer, rv = reverse primer.

To perform the PCRs, DNA was extracted from 100 μL of the resuspended BALF pellet or serum using the QIAamp DNA Micro kit (Qiagen GmbH, Hilden, Germany) according to the manufacturer’s instructions. RNA was extracted from 140 μL oral fluid or serum with the QIAamp Viral RNA Mini kit (Qiagen GmbH, Hilden, Germany). DNA was eluted in a 50 μL volume of buffer, and RNA in 60 μL buffer.

The PCR for the detection of PCS [9] was performed as previously described. For PCV2 [10], PRRSV [6], HPS [11] and MH [3] published protocols were modified.

### PCV2, HPS and MH PCRs

The PCR reaction master mixture for all PCR reactions consisted of 10 μL HotMasterMix (5Prime, Eppendorf, Vienna, Austria), 0.4 μM of each primer, 1 (HPS, MH) to 2 (PCV2) μL template DNA and distilled water to a total volume of 25 μL. The PCR reactions were started with a first heat denaturation step at 94°C for 2 min, followed by 40 cycles of heat denaturation at 94°C for 30 s, primer annealing at 55°C for 30 s (HPS, MH) or 60°C for 30 s (PCV2) and DNA elongation at 72°C for 1 min. Finally, a last DNA elongation step was carried out at 72°C for 10 min.

### PRRSV RT-PCR

The PCR was performed as previously described [6]. The only difference to the published PRRSV RT-PCR protocol was that in the present study the RNA extract was used undiluted.

### SIV RT-PCR

The reverse transcription (RT-) PCR assay of the RNA samples was carried out using the Qiagen^®^ OneStep RT-PCR kit (Qiagen, Vienna, Austria) according to the manufacturer’s instructions. The PCR reaction was started with a reverse transcription step at 50°C for 30 min, followed by an initial PCR activation step at 95°C for 15 min, 40 cycles of heat denaturation at 94°C for 30 s, primer annealing at 60°C for 30 s and DNA elongation at 72°C for 1 min. Finally, a last DNA elongation step was carried out at 72°C for 10 min.

A 10 μL sample of each PCR product was analyzed by gel electrophoresis in a Tris acetate-EDTA-2% agarose gel. Subsequently, the agarose gel was stained with ethidium bromide and bands were visualized using BioSens gel imaging system software (GenXpress, Wiener Neudorf, Austria). PCR products that had the expected product size (Table 1) were regarded to indicate positive results.

PCR products from 4 HPS positive samples per farm were extracted using the MinElute PCR Purification kit (Qiagen, Vienna, Austria) and were submitted for Sanger DNA sequencing (Microsynth, Balgach, Switzerland). The nucleotide sequences were analyzed using a BLAST search of the GenBank database. The sequences were assigned to serovars and their virulence was estimated according to Rubies et al. 1999 [12].

### Statistical analysis

A one-way ANOVA was calculated to identify significant differences between the mean PCS concentrations (log10 genome copies/mL BALF) on the two farms at the specific time points (IBM SPSS Statistics Version 24). The individual result was the statistical unit and the significance level was set at p = 0.05.

## Results

The quantitative PCS nucleic acid detection results, as well as the antibody profiles of the two farms are shown in Table 2. On Farm A, 5 of 8 suckling piglets in the 1^st^ week of life were positive and had a mean PCS burden of 4.6 log10 genome copies/mL BALF. In the groups S2 and W1, all pigs tested were positive and the concentrations were 7.9 and 8.3 log10 genome copies/mL BALF on average, respectively. In group W2, only one pig was positive and the PCS concentration was 0.9 log10 genome copies/mL BALF. In group F, the fungus could not be detected. Farm B had a different profile. The pigs were negative until the 3^rd^ month of life. In group W2, 4 of 8 pigs and in group F, 8 of 15 pigs were positive. Group W2 had a mean burden of 3.9 log10 genome copies/mL BALF and group F one of 4.0 log10 genome copies/ml BALF. Comparing the fungal loads per age class on the two farms, there was a significant difference (p = 0.030).

**Table 2:** Concentrations of *Pneumocystis* genomic DNA and concentrations of antibody against *Pneumocystis*.

	Method		S1	S2	W1	W2	F	SOW
**Farm A**	**qPCR**	**Mean quantity (log10 copies/mL BALF)**	**4.6**	**7.9**	**8.3**	**0.9**	0.0	n. a.
		**Standard deviation (log10 copies/mL BALF)**	3.8	3.2	1.1	2.6	0.0	n. a.
		**Median value (log10 copies/mL BALF)**	6.9	8.9	8.2	0.0	0.0	n. a.
		**Minimum quantity (log10 copies/ml BALF)**	0.0	0.0	6.8	0.0	0.0	n. a.
		**Maximum quantity (log10 copies/ml BALF)**	7.8	9.5	10.3	7.3	0.0	n. a.
		**Number of positive samples**	**5/8**	**7/7**	**8/8**	**1/8**	0/14	n. a.
	**ELISA**	**Mean OD**	0.48	0.31	0.26	0.55	0.66	1.30
		**Standard deviation**	0.18	0.24	0.10	0.36	0.34	0.76
		**Number of positive samples**	**7/8**	**3/8**	**2/8**	**6/8**	**12/15**	**16/16**
**Farm B**	**qPCR**	**Mean quantity (log10 copies/mL BALF)**	0.0	0.0	0.0	**3.9**	**4.0**	n. a.
		**Standard deviation (log10 copies/mL BALF)**	0.0	0.0	0.0	4.2	3.9	n. a.
		**Median value (log10 copies/mL BALF)**	0.0	0.0	0.0	3.7	6.8	n. a.
		**Minimum quantity (log10 copies/ml BALF)**	0.0	0.0	0.0	0.0	0.0	n. a.
		**Maximum quantity (log10 copies/ml BALF)**	0.0	0.0	0.0	8.2	8.4	n. a.
		**Number of positive samples**	0/3	0/13	0/8	**4/8**	**8/15**	n. a.
	**ELISA**	**Mean OD**	0.47	0.26	0.18	0.16	0.48	0.83
		**Standard deviation**	0.24	0.31	0.19	0.11	0.26	0.53
		**Number of positive samples**	**2/3**	**2/13**	**1/8**	**1/8**	**10/15**	**16/16**

S1 = suckling piglets in the 1^st^ week of life, S2 = suckling piglets in the 3^rd^ week of life, W1 = weaned piglets in the 2^nd^ month of life, W2 = weaned piglets in the 3^rd^ month of life, F = fattening pigs in the 4^th^ month of life; SOW = sows; n. a. = not analyzed; OD = optical density.

There were antibody positive pigs in all age classes. All sows and nearly all piglets in group S1 on both farms had anti-PCS antibodies. The number of antibody positive piglets decreased in both farms until the 3^rd^ week of life. On Farm A, there was an increase of the number of antibody positive pigs in group W2. The proportion number of antibody positive pigs was similar in each age class up to those in class F. On Farm B, groups of pigs between the 3^rd^ week and 3^rd^ month of life had only single pigs with anti-PCS antibodies, while in group F 2/3 of the animals tested were antibody positive.

The prevalences of the pathogens on Farm A were 47% for PCS, 15% for PRRSV, 15% for SIV, 87% for HPS, and 64% for MH. On Farm B, 26% of the pigs were positive for PCS, 47% for SIV, 89% for HPS, and 32% for MH. PCV2 could not be detected on either farm and Farm B was negative for PRRSV. PCS and the other pathogens investigated except MH on Farm A, SIV on Farm B and HPS on both farms were detectable age class-related (Table 3). Sanger DNA sequencing of PCR products from 4 samples from Farm A had a 100% identity to the outer membrane protein gene sequence in GenBank accession no. CP009158, a HPS strain assessed to be virulent with a high probability (serovar 12), whereas the sequences of products obtained from samples from Farm B had 99% identity to the outer membrane protein gene sequence of the non-virulent HPS strain HM172009 (serovar 9).

**Table 3:** Associations between *Pneumocystis* and the other respiratory pathogens in different age classes of pigs.

	Pathogen	Total	S1	S2	W1	W2	F
**Farm A**	***Pneumocystis carinii* f. sp. *suis***	21/45	5/8	7/7	8/8	1/8	0/14
	**PCV2**	0/47	0/8	0/7	0/8	0/8	0/14
	**PRRSV EU+US**	7/47	7/8	0/7	0/8	0/8	0/14
	**SIV**	6/42	0/4	0/7	0/8	0/8	6/15
	***Haemophilus parasuis***	39/45	8/8	7/7	7/8	7/8	10/14
	***Mycoplasma hyopneumoniae***	29/45	3/8	2/7	5/8	6/8	13/14
**Farm B**	***Pneumocystis carinii* f. sp. *suis***	12/47	0/3	0/13	0/8	4/8	8/15
	**PCV2**	0/47	0/3	0/13	0/8	0/8	0/15
	**PRRSV EU+US**	0/47	0/3	0/13	0/8	0/8	0/15
	**SIV**	16/34	0/1	3/6	0/8	4/6	9/13
	***Haemophilus parasuis***	42/47	1/3	11/13	8/8	8/8	14/15
	***Mycoplasma hyopneumoniae***	15/47	0/3	0/13	3/8	7/8	5/15

S1 = suckling piglets in the1^st^ week of life, S2 = suckling piglets in the 3^rd^ week of life, W1 = weaned piglets in the 2^nd^ month of life, W2 = weaned piglets in the 3^rd^ month of life, F = fattening pigs in the 4^th^ month of life, dark grey: ≥ 50% of the pigs were positive, light grey: < 50% positive.

On Farm A, the suckling piglets were infected with PCS and PRRSV in the 1^st^ week of life. While piglets in group S2 were PRRSV negative, PCS infection was detected in pigs up to 3 months old. The pigs on Farm A were also infected with a virulent HPS strain and MH. In pigs that were 4 months old, SIV infection was detectable. On Farm B, pigs in groups S1, S2, and W1 were PCS negative. Half of the samples in age class S2 were positive for SIV and nearly all pigs were infected with a strain of HPS predicted to be avirulent. In group W1 no SIV nucleic acid could be detected. In pigs aged 3 months or older, SIV infection was detected and these age classes were also PCS and MH positive.

## Discussion

This study is the first to describe the PCS nucleic acid and antibody profiles of two Austrian farrow-to-finish farms. It has to be considered that this is a cross-sectional study and results only reflect the present pathogen status in the specific age classes, but not a steady state. Nevertheless, this study gives a first impression of possible PCS infection patterns and associations with other lung pathogens. On both farms the course of PCS infection was clearly visible, but there were differences in the onset and the PCS burden. On Farm A PCS nucleic acid could be detected in the suckling piglets, while the infection was not detected in pigs under 3 months of age on Farm B. Kureljušić et al. (2016) [3] detected higher prevalences in suckling piglets than in weaned or fattening pigs, but PCS infection does not appear to be exclusively age class-related. On Farm B, all samples from the younger age classes (S1, S2 and W1) were PCS negative and PCS nucleic acid was only detectable when other pathogens were detectable. The mere detection of the fungus coincidentally with other lung pathogens is no proof for its impact on multifactorial caused respiratory diseases. Nevertheless, immunosuppressive conditions because of systemic infection with human immunodeficiency virus (HIV) or local co-infections of the respiratory tract are a pre-condition for lung disease caused by *Pneumocystis jirovecii* in humans [2,13]. CD4+ T cells are critical for immunological defence against *Pneumocystis*, because they are essential for memory-cell functions that coordinate host inflammatory responses responsible for the elimination of the organism. A second function of specific CD4+ T cell classes is the regulation of cytokine production and cytotoxic activity [14]. *Pneumocystis* pneumonia is more often observed when CD4+ T cell numbers are reduced [15] or when these cells are completely absent [16]. In contrast, CD8+ T cells can either be protective or detrimental, depending on their cytokine-associated phenotype [14]. The second difference between the PCS nucleic acid profiles was the higher burden on Farm A. Suckling piglets are probably more susceptible to severe infections and PCS proliferation because of their functionally immature immune system [17]. Immunity to PCS seems to be an interaction between humoral and cell-mediated components. Innate immune cells and associated cytokine and chemokine mediators are responsible for recognizing and clearing the organism, and for activating long-term adaptive responses. These adaptive immune responses are also critical for supporting the innate immune responses as well as for maintaining long-term memory protection [14]. On both farms, the proportion of animals serologically positive for PCS increased with age, and all sows were seropositive. It is not known how long antibodies are detectable after infection in pigs, but there is no consistent correlation between increases in antibody levels and time of diagnosis of *Pneumocystis* infection in humans [18]. In healthy humans, the seroprevalence for PCS can be found to be as high as 58% [19]. This implies that many humans have contact with this agent but are able to eliminate *Pneumocystis*. Depending on the test system used the prevalences of seropositivity can be high, but tests generally fail to distinguish active infection from previous exposure [20]. It remains unclear whether a single infection with PCS induces stable, long-lasting antibody titers or whether consecutive reinfection is necessary for prolonged seropositivity. In this study, the sows had higher OD values than the other age classes. Although the ELISA does not allow quantification of antibody activities, it can be assumed that high OD values correlate with high anti-PCS antibody titers, which might be a result of the pathogen, boosting the adaptive immune response. The clinical relevance of these findings still has to be investigated, but the establishment of dependable serological tests is urgently needed as it will allow the diagnosis of *Pneumocystis* pneumonia using specimens that can be collected non-invasively and inexpensively [21].

Nearly all suckling piglets had anti-PCS antibodies in the 1^st^ week of life, which were most probably maternal antibodies derived from colostrum. The antibody levels had decreased by the 3^rd^ week of life, when only 1/6 (Farm B) to 1/3 (Farm A) of the sampled pigs were antibody positive. Maternal antibodies are the main source of protective humoral immune responses during the first weeks of life and in pigs they begin to wane by about 5 weeks of age [22]. It is unknown if reduced colostrum intake, as well as a lack of anti-PCS antibodies in the colostrum, increases the risk of clinical pneumocystosis. Despite of the presence of maternal antibodies on Farm A, piglets in the 1^st^ week of life were not protected against PCS infection. Thus, maternal antibodies may not be sufficiently protective. In our study, maternal antibodies appeared to wane rapidly, the PCS burden increased and nucleic acid was detectable at concentrations of 7.9–8.3 log10 between 3 weeks and 2 months of life. An active humoral immune reaction could not be detected until 3 months after birth in the majority of piglets examined on Farm A, when 6 of 8 pigs had anti-PCS antibodies. On Farm B, anti-PCS antibodies were detected in 2/3 of the fattening pigs one month after infection, even though their PCS burden was significantly lower.

Prior to this study, Express Biotech International (Frederick, MD, USA) provided ELISA tests for the detection of antibodies against rat and mouse *Pneumocystis*. The XpressBio *Pneumocystis carinii* swine ELISA was established and validated with our samples on request. The results of the present study show clearly that the ELISA is a practical alternative for farm screening. The collection of blood samples is easier than BALF sampling because pigs do not have to be anesthetized. Furthermore, ELISA procedures can be easily established and are a standardized, reliable, fast and cheaper alternative to direct pathogen detection methods.

The second aim of this study was the investigation of possible associations with other respiratory pathogens. Results of prior studies suggested causative relation hips [3,6]. On Farm A, pigs were positive for PRRSV, HPS and MH concurrently with the PCS infection in the 1^st^ week of life. A vertical infection with PRRSV could not be excluded, but an increase in preweaning mortality might have been expected, if this was the case. In the past, the farm had had to deal with chronic PRRSV infections. The positive piglets could be the result of a recurrent acute infection. Unusually, the older age classes were not viremic. PRRSV replicates in macrophages and dendritic cells located in the tonsils, upper respiratory tract and lungs, and viremia starts 12 hours post-infection [23]. In our study, samples were collected from one week old piglets. Pigs are extremely susceptible to aerosol infection [23] and, for this reason, a horizontal transmission is a more likely explanation. A common finding on PRRSV positive farms is that there is usually a higher prevalence of the virus in serum of weaned pigs, which was not seen on this farm. According to infectivity, transmission rate and virulence there are enormous differences between PRRSV strains. This strain might be less virulent with a relatively short viremic phase. It can be assumed, that pigs were still persistently infected with virus present in lymphatic tissue.

Co-infections in PCS positive pigs have been reported previously. Kim et al. (2011) [24] found that 12.8% of PCS positive pigs were co-infected with PRRSV and 48.7% were co-infected with PCV2 and PRRSV. Furthermore, they identified three PCS positive pigs with concurrent bacterial infection. In a study by Weissenbacher-Lang et al. (2016) [6], PRRSV was detected in 7% and MH in 29% of PCS positive animals. In suckling piglets, significantly more samples were positive for PCS in combination with viruses, such as PCV2, PRRSV, and torque teno sus virus, whereas older pigs had polymicrobial co-infections with these viruses as well as bacterial pathogens such as *Bordetella bronchiseptica*, *Pasteurella multocida*, and MH. The distribution of PCS positive samples on the two farms differed significantly. On Farm A PCS infection occurred early in life and the suckling piglets were infected in the 1^st^ week of life. PCS infection was accompanied by PRRSV, a virulent strain of HPS and MH. It has been shown previously that co-infection results in higher PRRSV viral loads and more severe lung lesions than in pigs infected with only one of these two pathogens [25]. Similarly, pigs co-infected with PRRSV and HPS have a higher HPS load than pigs only infected with HPS [26]. Palzer et al. (2015) [27] found that PRRSV positive pigs were more likely to be HPS positive. On Farm B, PCS infection occurred later, at 3 months of age, and was accompanied by infection with SIV, MH and a non-virulent strain of HPS. There appears to be minimal interaction between SIV and MH [28], and synergisms between SIV and HPS have not been well investigated [29,30].

Our results show that the PCS profiles can vary between farms. *Pneumocystis* is an opportunistic fungus and its proliferation is triggered by immunosuppression. Suckling piglets are probably more susceptible to an infection, can be infected very early in life and can have higher PCS burdens but, nevertheless, PCS may also contribute to respiratory disorders in older pigs. In the present study, PCS proliferation in pigs on both farms may have been triggered by co-infections. A more detailed characterization of the courses of infection of these respiratory pathogens, as well as their interaction with PCS, could be carried out by increasing the number of pigs sampled, comparison of more farms and using quantitative detection techniques.
